# Size-Based Hydroacoustic Measures of Within-Season Fish Abundance in a Boreal Freshwater Ecosystem

**DOI:** 10.1371/journal.pone.0124799

**Published:** 2015-04-15

**Authors:** Riley A. Pollom, George A. Rose

**Affiliations:** Centre for Fisheries Ecosystems Research, Fisheries and Marine Institute of Memorial University of Newfoundland, St. John's, Newfoundland and Labrador, Canada; Pacific Northwest National Laboratory, UNITED STATES

## Abstract

Eleven sequential size-based hydroacoustic surveys conducted with a 200 kHz split-beam transducer during the summers of 2011 and 2012 were used to quantify seasonal declines in fish abundance in a boreal reservoir in Manitoba, Canada. Fish densities were sufficiently low to enable single target resolution and tracking. Target strengths converted to log_2_-based size-classes indicated that smaller fish were consistently more abundant than larger fish by a factor of approximately 3 for each halving of length. For all size classes, in both years, abundance (natural log) declined linearly over the summer at rates that varied from -0.067.day^-1^ for the smallest fish to -0.016.day^-1^ for the largest (R^2^ = 0.24–0.97). Inter-annual comparisons of size-based abundance suggested that for larger fish (>16 cm), mean winter decline rates were an order of magnitude lower (-0.001.day^-1^) and overall survival higher (71%) than in the main summer fishing season (mean loss rate -0.038.day^-1^; survival 33%). We conclude that size-based acoustic survey methods have the potential to assess within-season fish abundance dynamics, and may prove useful in long-term monitoring of productivity and hence management of boreal aquatic ecosystems.

## Introduction

In freshwater ecosystems, the relative abundance and dynamics of size-classes of organisms are keys to understanding energetics and production [1]. In these ecosystems, predators and prey often display distinct and predictable body size ratios [2] that may reflect trophic levels [3], [4]. A positive relationship between body size and trophic level is likely in aquatic systems because fish are morphologically constrained by gape limitation, and hence limited to prey within a specific size range [5], [6].

Aquatic net sampling methods have been used to examine size structure among fishes, but are typically too size-selective to represent all size classes without major bias [7], [8]. Net surveys also cause unintentional mortality of fish and crustaceans and are expensive when sampling large systems [9].

Hydroacoustic methods enable more comprehensive, non-lethal and cost-effective assessments of the pelagic portion of the aquatic environment [10], with potential quantification of the distribution and abundance of organisms ranging in size from zooplankton to large predatory fish at high resolution [11]. Most applications of acoustics to date have been based on echo integration techniques that do not depend on isolation of single organisms in the acoustic beam [12]. Size-based acoustic measures have been less successful, as densities often exceed a threshold above which echoes cannot be isolated as single targets [13]. There may also be bias in the availability of size classes to the acoustic beam in the so-called surface and bottom exclusion dead zones [14], or at increasing ranges from the acoustic beam axis. These biases may be minor, however, in shallow boreal freshwater ecosystems surveyed at relatively high acoustic frequencies where the exclusion zones are small and the majority of fish targets are encountered individually [15].

For fisheries management, indicators of within-season abundance and mortality are important to setting catch restrictions and monitoring the current state of the fisheries ecosystem. Moreover, within-season dynamics may enable better predictions of ecosystem productivity across systems and in coming years (e.g., strong or weak recruitment, low or high mortality). In theory, natural and fishing mortality in aquatic ecosystems should be reflected in seasonal declines in abundance and increases in the slope of size-frequency data [16–18]. In addition, inter-annual dynamics of various size classes of fish should be reflected in surveys. There have been few attempts, however, to test whether acoustic survey methods are sufficiently sensitive to measure such dynamics [12].

The main objective of this study was to test if size-based hydroacoustic surveys could describe seasonal and inter-annual variation in fish abundance in a boreal freshwater ecosystem. Expectations of theory were tested, namely that: 1) larger fish would be less abundant than small fish; 2) smaller fish would decline at rates greater than would larger fish over seasons; and 3) that inter-annual abundances of size classes would be consistent with mortality expectations from year to year. Based on these findings, it is suggested that size-based acoustic methods could make a substantial contribution to the management and long-term monitoring of freshwater ecosystems.

## Methods

### Study Area

Lac du Bonnet is a hydroelectric reservoir on the Winnipeg River in southeastern Manitoba (50° 22′N 95° 55′W), located between the Seven Sisters and MacArthur dams, two large run-of-river hydroelectric operations (no specific permissions were required for these locations/activities as Lac du Bonnet is public access). The area is moderately developed, with several small communities as well as many summer homes and cabins along its banks, and is heavily used for recreational boating and fishing.

**Fig 1:**
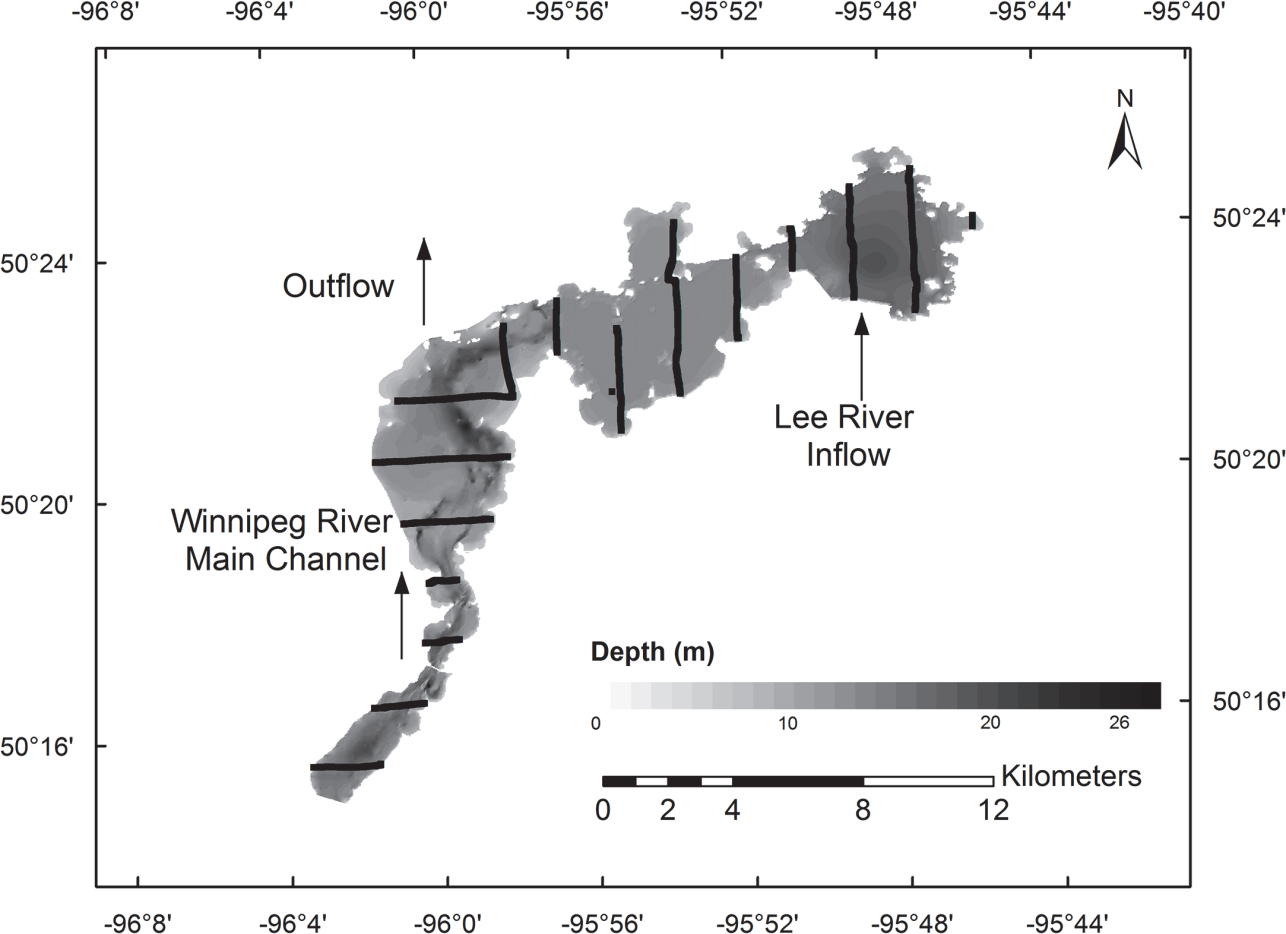
Map of Lac du Bonnet. Detailed map of Lac du Bonnet with depth contours and GPS-recorded transect lines from Survey 2. Note that survey tracks were sequentially offset within years in order to cover the entire reservoir over each study period.

The Lac du Bonnet reservoir comprises three distinct basins (Fig 1). The largest basin is the flooded main channel of the Winnipeg River, which runs south to north during this stretch and covers approximately 38.5km^2^. Other than the main channel, which reaches a maximum depth of 26 m, this basin generally has depths of <5m. The middle basin is deeper (~10–12m), covers 27 km^2^ and is distinguished by several large bays with small creek tributaries and wetlands. The northeast basin is deepest (15–20m) and covers close to 18.5 km^2^. This latter basin is fed by the lower-flow Lee River from the south and has mostly steep rocky banks.

The Lac du Bonnet reservoir has a maximum depth of 30 metres, with a summer temperature of approximately 22°C. Flow levels in the reservoir are sufficient to prevent stratification and thus summer temperatures are relatively constant throughout all depths and locales within the reservoir. The waters of Lac du Bonnet can be classified as mesotrophic to eutrophic, with high turbidity (a secchi depth of ~1.2m) and occasional phytoplankton blooms in late summer (authors’ personal observations). The system’s catchment area lies in the transition zone between the aspen parkland with its underlying sedimentary substrate and the boreal forest on the igneous and metamorphic rocks of the Canadian Shield. The reservoir is characterized by a diverse array of large fish species that include walleye (*Sander vitreus*), sauger (*Sander canadensis*), northern pike (*Esox lucius*), lake whitefish (*Coregonus clupeaformis*) and burbot (*Lota lota*), in addition to smaller cisco (*Coregonus* spp.) and shiners (*Notropis* spp.)(authors’ personal observations, [19]). In all, 27 fish species were observed during summer net surveys conducted concurrently in the pelagic and littoral zones of the reservoir in 2011 and 2012 for an adjacent study (D. Boisclair *Université de Montréal*, personal communication). No endangered or protected species were handled or disturbed during this study.

### Acoustic Surveys

Eleven daytime hydroacoustic surveys taking 6–8 hours each were performed in July and August of 2011 and 2012 using a BioSonics split-beam DTX echosounder (BioSonics, Seattle, WA, USA) with 200, 430, and 1000 kHz transducers (only the 200 kHz data are used here; this transducer had a half-power beam angle of 6.5° and transmitted at 6 pings.s^-1^) (Table 1). All acoustic data were georeferenced with an integrated GPS (Garmin 17xHVS, Garmin Ltd., Olathe, KS, USA) and collected using Visual Acquisition Software version 6.0.2 (BioSonics Inc., Seattle, WA, USA). Transducers were deployed on a custom-built aluminum arm mounted off the port side of a 17′ Boston Whaler with a foam-cored hull and 90 hp four-stroke engine to reduce noise [20]. During surveys, the transducer faces were between 40 and 50 cm below the water surface. Transducers were calibrated using tungsten carbide spheres to well-established standards [21]. Parallel straight-line transects were run at approximately 9–11 km.h^-1^ (5–6 knots) along the short axis of each basin (Fig 1), as close to the shoreline as possible (typically to ~2m depth on the sounder) [11], [22]. The first transect position was chosen at random, thereafter transects were spaced 1850m apart spanning the entire reservoir. After the first survey, transects were offset by approximately 150 m in each subsequent survey to provide more comprehensive bathymetry and spatial data across the reservoir.

**Table 1:** Hydroacoustic Surveys conducted over the study period at Lac du Bonnet.

Survey Number	Date	Total Transect Length (km)	Coverage
1	July 26th, 2011	37.39	4.08
2	August 2nd, 2011	40.68	4.44
3	August 3rd, 2011	38.41	4.19
4	August 15th, 2011	35.23	3.84
5	August 18th, 2011	40.91	4.46
6	August 27th, 2011	35.15	3.83
7	July 28th, 2012	36.39	3.97
8	August 2nd, 2012	40.23	4.39
9	August 8th, 2012	35.74	3.90
10	August 11th, 2012	39.66	4.33
11	August 24th, 2012	41.39	4.52

Survey coverage is defined as the total transect length divided by the square root of the reservoir area [11].

### Data Analysis

Echograms from all transects were scrutinized then edited using Echoview version 5.0 (Myriax Inc., Hobart, TAS, Australia). The lakebed was delineated using a smoothing filter on the best bottom candidate line picks, with manual edits where necessary to include data as close to the lake bottom as possible while excluding the strong signal associated with the substrate. A surface line was imposed at a depth below the majority of surface noise (a minimum of 2 metres was excluded—actual values varied among surveys depending on wind conditions—there was no trend in the exclusion over time). Manual edits removed minor extraneous noise.

A single target detection algorithm identified individual fish and provided target strength (TS_dB,_ hereafter TS) data along each transect. Detection parameters included a TS threshold of -52.6 dB (equivalent to a freshwater fish of 4 cm length with a swim bladder according to the model of Love [23]), a pulse length determination level of -6 dB, and minimum and maximum normalized pulse lengths of 0.7 and 1.50, respectively (Table 2). Attempts to decrease the threshold below -52.6 dB were abandoned as a consequence of uncertainties about separation of fish without clouds of large zooplankters that were abundant in the reservoir (unpublished data). We allowed a maximum beam compensation that was larger than typically used in studies of TS, which increased the effective beam volume and number of fish that could be measured. Tests comparing narrower and wider allowable beam compensations indicated the expected increase in measured targets with larger compensation, but no significant differences in TS characteristics, although a small bias towards larger targets further from the beam axis with increased allowed compensation was evident in some samples. Initial tests of single target and integrated densities indicated that Sawada’s N_v_ never exceeded 0.01, hence single target data were considered to be unbiased [13].

**Table 2:** Single target settings.

**Single Target Settings**	
TS threshold	-55 dB
Pulse length determination level (PLDL)	6 dB
Minimum normalized pulse length	0.7
Maximum normalized pulse length	1.5
Beam compensation model	BioSonics
Maximum beam compensation	15 dB
Maximum standard deviation of minor-axis angles	1.2
Maximum standard deviation of major-axis angles	1.2
**Fish Track Detection Properties**	
Minimum number of single targets	1
Minimum number of pings in track	1
Maximum gap between single targets	2

Single target detection settings and fish track detection properties used in Echoview 5.0 ((Myriax Inc., Hobart, TAS, Australia) with BioSonics DTX 200 kHz split-beam echosounder (BioSonics Inc., Seattle, USA).

Tracking of individual fish was based on sequential TS echoes and track acceptance parameters were designed to include all single targets but to group sequential TS values presumed to come from single fish (Table 2). Small ranges and relatively rapid survey speeds led to most tracks being single TS echoes. For tracks with n>1 the maximum TS was used. Tracks were manually edited where necessary to limit perceived grouping errors in rare cases where two or more fish were close together. The so-called acoustic near bottom dead zone ranged from approximately 0.5–0.6 m and undoubtedly resulted in missing some benthic fish. Any bias was thought to be constant, however, as all surveys used here were run during daylight hours (night-time surveys were also attempted but plankton was sufficiently thick to make isolation of small fish targets problematic—unpublished data).

Fish tracks were separated into five log_2_ scale size classes based on the target-strength length relationship outlined in [23]. In its modified form taking into account the 200 kHz frequency at which the data were collected, Love’s equation indicates:

$$Fish\;Length\;\left(cm\right)=10^{((TS}{{}_{dB}}^{+64.09)/19.1)}$$

These size classes span a range from large predatory fish important to recreational fisheries to 4 cm forage fish and juveniles that would be prey for both medium and large fish (size classes of 4–7.9 cm, 8–15.9 cm, 16–31.9 cm, 32–63.9 cm, and >64 cm). Love’s equation may not be entirely applicable to all fishes measured in this study, but it provided a consistent relative basis to scale the acoustic TS to biological size of the surveyed fish community. Counts of fish of each size class were then tabulated for each survey, and their natural logarithms plotted against size class. Linear regressions were performed using XLSTAT Version 2014.2.03 (Addinsoft, Belmont, MA, USA).

## Results

**Fig 2:**
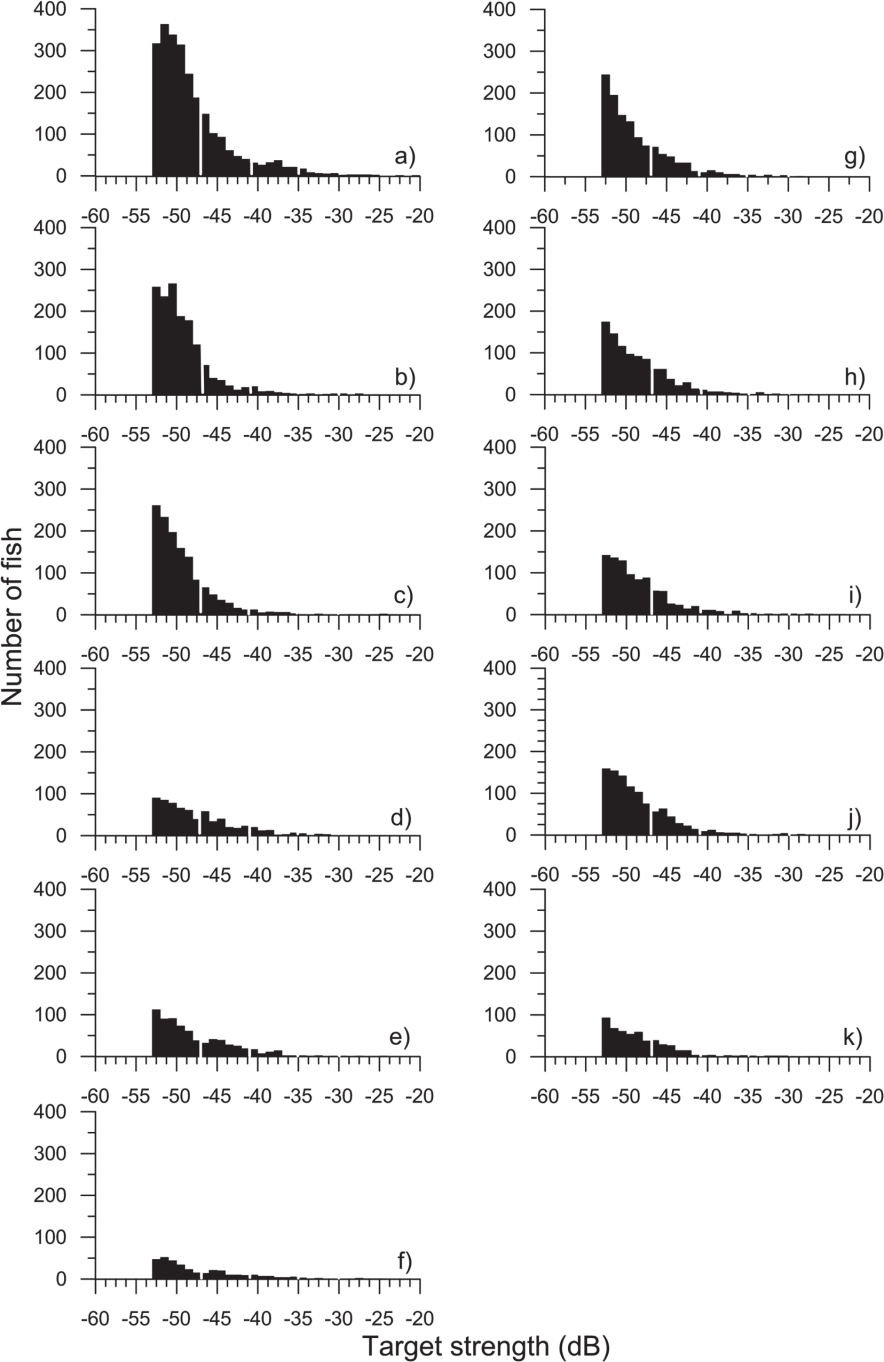
Acoustic target strength (dB) counts of fish tracks. Counts of fish tracks for the different size classes obtained during the 11 surveys of Lac du Bonnet in 2011 and 2012. The white dividing lines represent the breaks among assigned size classes.

**Fig 3:**
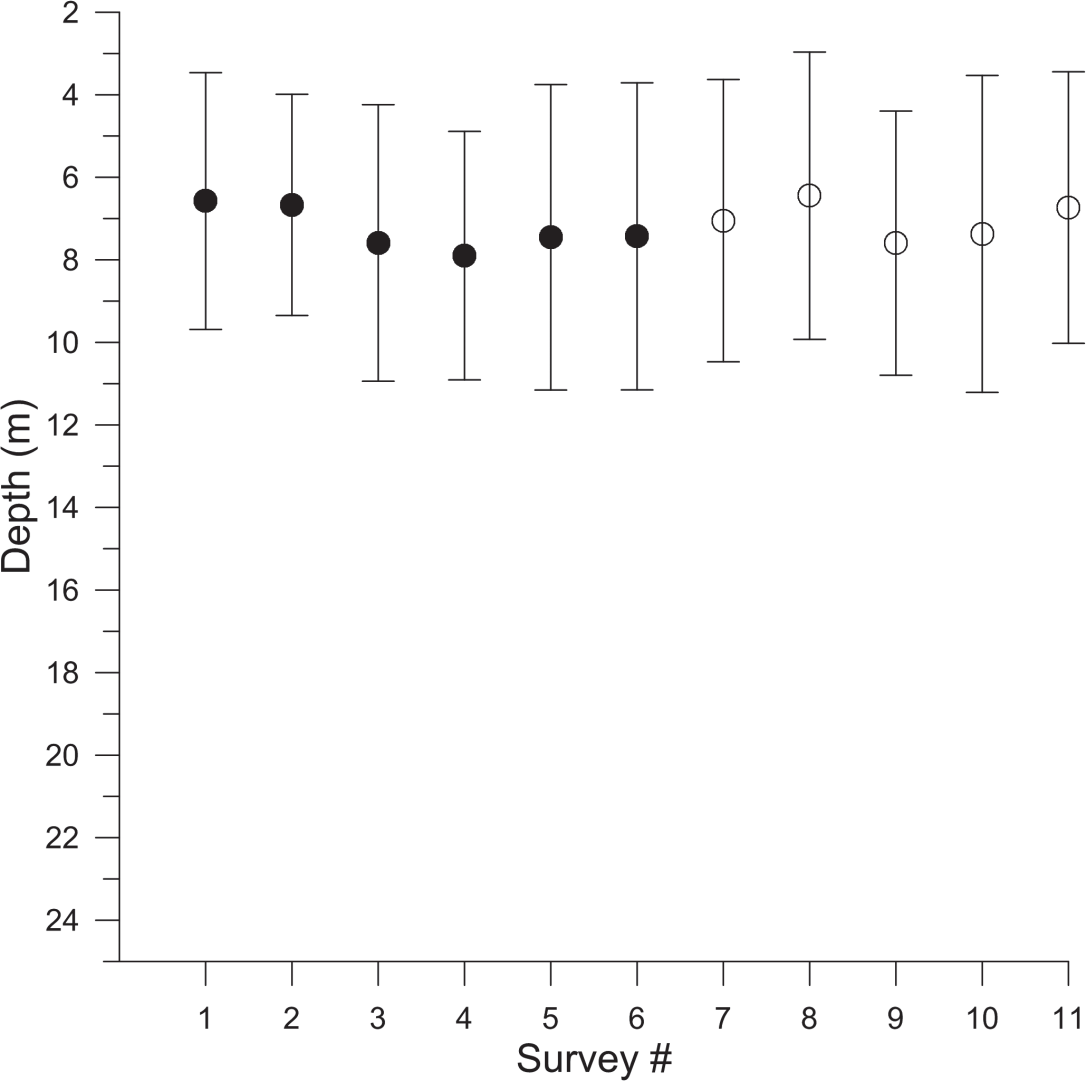
Average depth distribution of targets. The average depth distribution and 95% CI of all targets for each acoustic survey of Lac du Bonnet in 2011 (closed circles) and 2012 (open circles). There was no trend in target depth.

The acoustically-derived fish counts surveyed at Lac du Bonnet during the summers of 2011 and 2012 indicated that TS distributions ranged from approximately -52.6 dB (threshold limited) to a very few tracks that measured in the low -20 dB range (Fig 2). All survey counts were strongly skewed right. There was little indication of a shift of counts from one size class to the next largest during the study periods (33 days in 2011 and 28 in 2012), but a decline in all classes over the summer was evident. There was little indication of recruitment to the smallest size class in either year. Fish were on average at similar depths in all surveys (Fig 3).

**Fig 4:**
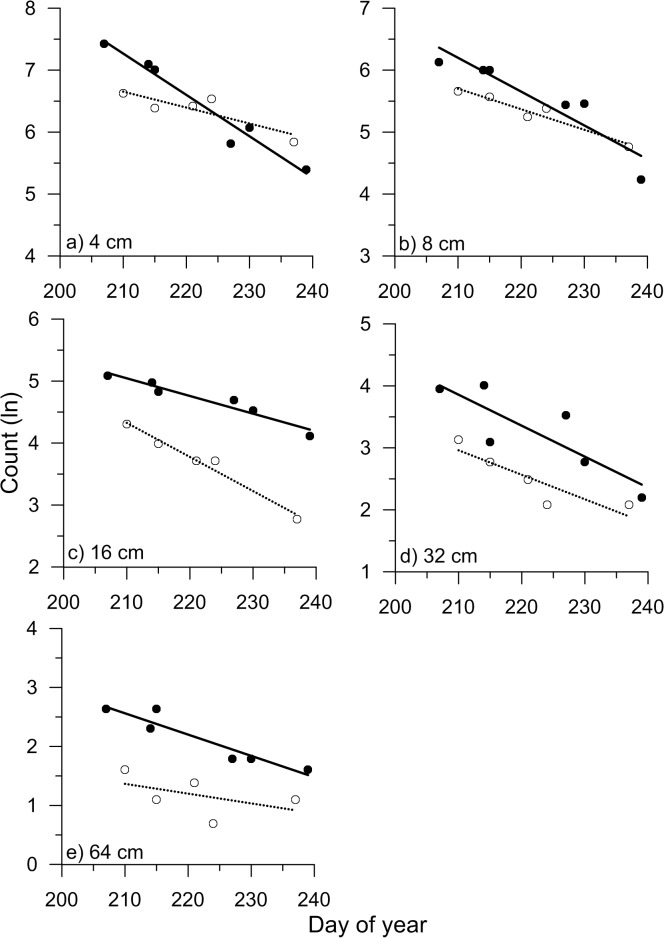
Counts of fish from acoustic surveys. Natural logarithmic counts of 5 target strength size classes (a-e representing size groups 4, 8, 16, 32 and 64 cm, respectively) from the 11 acoustic surveys carried out on Lac du Bonnet in 2011 (closed circles) and 2012 (open circles). Statistics of lines are given in Table 3.

Natural log-transformed counts of all size classes of fishes declined linearly throughout the summer season (Fig 4). All surveys showed abundance declining between adjacent size classes by approximately 1 natural logarithm unit or a factor of approximately 3 (e.g., size 4 cm fish were on average approximately 3 times as abundant as size 8 cm fish). Day of year explained between 74.7 and 97.4% of variation in seasonal abundance declines for the 5 size classes with the exception of the largest size class in 2012 which declined over the season but with greater variability among surveys (Table 3).

**Table 3:** Linear regression results of acoustic survey counts of fish for each target strength-upper limit of size class by year, with lower threshold of -52.6 dB.

Maximum Target Strength (dB)	Year	Ln Initial Count	Slope	p Value	R^2^
-46.85	2011	7.423	-0.067	0.001	0.96
-46.85	2012	6.624	-0.026	0.059	0.75
-41.05	2011	6.131	-0.055	0.008	0.86
-41.05	2012	5.659	-0.033	0.007	0.93
-35.35	2011	5.088	-0.029	0.002	0.94
-35.35	2012	4.304	-0.055	0.002	0.97
-29.6	2011	3.951	-0.050	0.034	0.72
-29.6	2012	3.135	-0.039	0.044	0.79
-23.85	2011	2.639	-0.036	0.005	0.89
-23.85	2012	1.609	-0.017	0.398	0.24

Assignment to size class based on [23]. Slope is an estimate of loss (mortality) rate.day^-1^.

**Fig 5:**
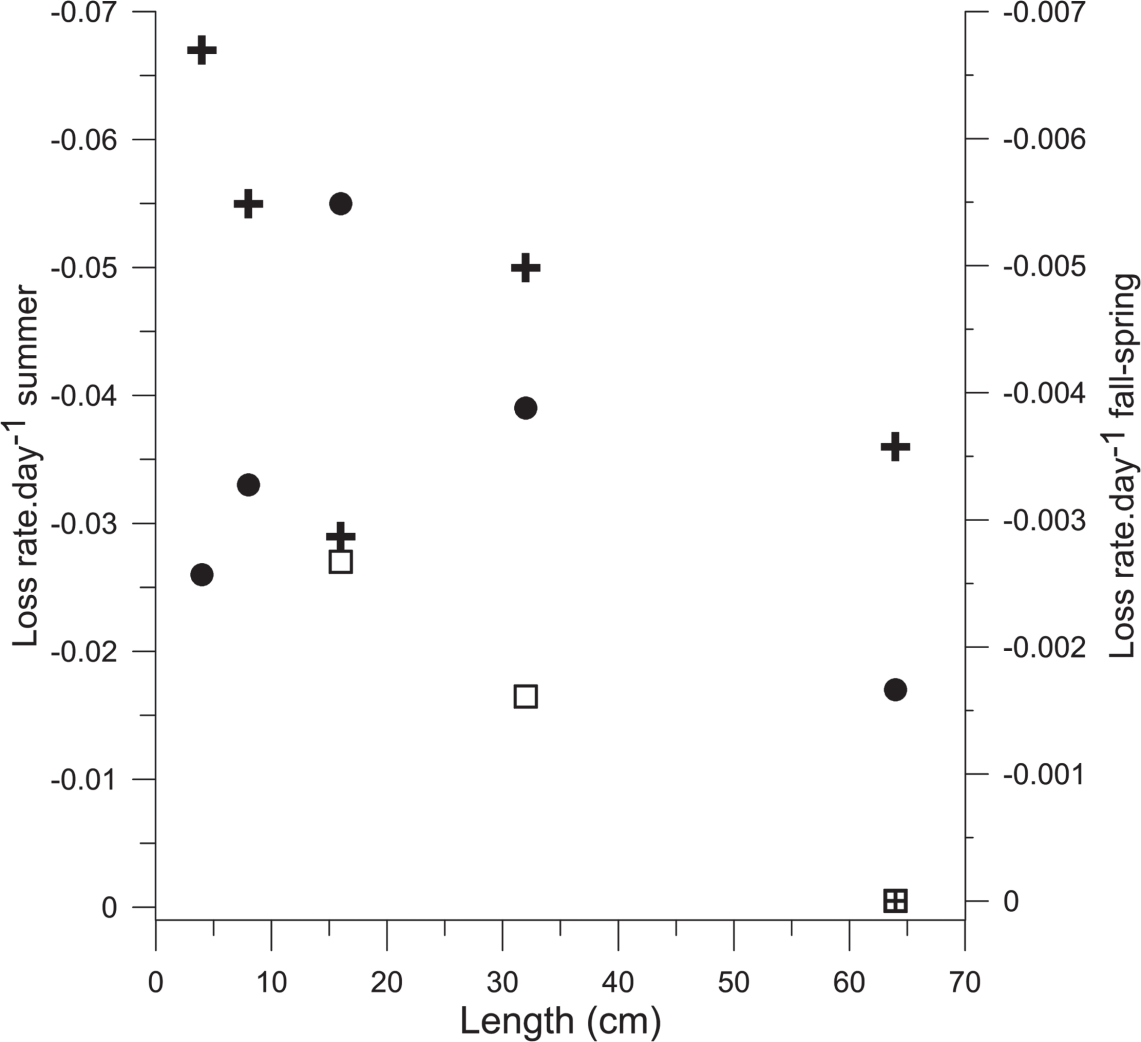
Daily declines in fish abundance. Daily loss rates (declines in counts) within the summer study periods for the 5 size classes in 2011 (crosses) and 2012 (circles), scale on left axis. Inter-annual loss rates from the last survey in 2011 to first survey of 2012 (squares) for size classes (>16cm) (64 cm class to same class) (crossed square), scale on right axis.

Instantaneous loss rates in the counts over both study periods were variable but generally lower with increasing fish size (Fig 5). Decline rates over the approximately 30-day study periods in both years ranged from -0.067 to -0.016.day^-1^. From the end of surveys in 2011 to the first survey in 2012, for the largest 3 size classes, loss rates ranged from approximately -0.0026 to near 0.day^-1^, an order or magnitude lower than during the summer study period. The smallest size classes were not considered because recruitment, not growth, was almost certainly the main factor in their abundance dynamics from year to year. Based on these data, the mean instantaneous loss in size classes (> 16cm) was approximately 0.001.day^-1^ over the approximately 11 months that were not surveyed, or a fall to summer survival of 71%. In contrast, summer survival during the present study period averaged 33% for the same large size classes. Loss rates of the largest 2 size classes of fish were considerably higher during the summer fishery than during the rest of the year (Fig 5).

## Discussion

The results obtained in this study indicate that size-based hydroacoustic assessments of boreal freshwater ecosystems have potential to enable monitoring of the density and abundance of various size classes of fish both within and between years. During the summer study periods the highest rates of loss occurred with the smallest fish and the lowest in the largest in both years, consistent with size-based and population dynamics theory (e.g. [24]). Comparisons of incremented size classes from 2011 to 2012 gave a similar result, but with much lower losses and higher apparent survival. The instantaneous loss rates are equivalent to estimates of mortality, which during summer is likely a result of the intensive recreational fishery, assuming little emigration from the survey area [25].

The data for the largest two size classes of fish are likely to be of most interest to the sport fishery (>-35.35dB target strength or 16 cm). Their decline, especially in 2011, was in line with the relatively high fishing pressure in Lac du Bonnet (Doug Leroux, Manitoba Conservation, Lac du Bonnet, Manitoba, personal communication). Summer mortality appeared to be of the order of that observed annually in walleye in heavily fished lakes in New York [26].

Although inter-annual comparisons can only be made over a single year with the present data, the apparently low losses of larger fish compared to those that occurred during the summer study periods suggest that the fishery is the main source of mortality of larger fishes in this ecosystem. In addition, the initial number of small fish (both 4 and 8 cm size groups) in 2011 was higher than in 2012, which suggests variable production (recruitment), and suffered a higher loss rate over the season, which is consistent with density-dependent mortality. The larger size classes do not show this effect. These findings must be considered preliminary given the limited comparisons, but nonetheless are at least suggestive that further information on population dynamics could be garnered from longer time series of such surveys.

Size-based measures have potential to provide a method to characterize ecological relationships among fish of multiple size classes, and perhaps trophic levels. Counts of individual fish of the various size classes were negatively related to body size, as metabolic theory and trophic transfer efficiency predict [27–30]. Previous studies have linked fish body size to trophic level on regional and global scales in marine systems [6], [31], as well as in temperate lakes in Ontario near Lac du Bonnet having similar fish communities [32]. This latter study however found body size predictions of δ^15^N-derived trophic level to be relatively weak, suggesting factors other than body size may also be involved. Additional research is recommended to further explore these potential relationships as they relate to fish productivity.

It was not clear when this study was planned that hydroacoustic methods could be successful in describing size-based fish communities in the study system. We found, however, that potential limitations, particularly densities too high to reliably extract single targets, were not encountered during this study (nor were they in exploratory companion studies of freshwater salmonid ecosystems in Newfoundland. Variations in TS and hence allocated size as a consequence of behavioural dynamics and variations in the cross-section aspect of fish might also make size-classes problematic, but the consistency of the present results suggest that any such variations did not systematically bias the results of this study.

We acknowledge several limitations of the present study. The work was conducted over two summers, which limited inter-annual comparisons. In addition, only acoustic targets with TS > -52.6 db (4 cm by Love’s equation [23]) were extracted, thereby excluding the smallest and likely most abundant organisms. It is very likely that counts of the smallest fish were underestimated relative to those of larger fish as a consequence of decreased signal to noise ratios. That there were more 8 cm fish counted at the start of the 2012 study than 4 cm fish at the end of the 2011 study is consistent with that interpretation. In addition, for the largest and perhaps more benthically-oriented fish, a negative bias could potentially exist with the near-bottom dead zone exclusion. It is equally likely, however, that any bias was constant over the summer, making the time series of relative abundance comparable.

## Conclusions

We conclude that size-based hydroacoustic methods have the potential to monitor seasonal and inter-annual fish mortality and provide information fundamental to the state of fisheries and freshwater ecosystems. These methods are essentially less intrusive, more cost-efficient and perhaps less biased than traditional net-based surveys, and could prove to be an effective tool for ecosystem-based, rather than single species-based fisheries management [33]. Further studies and longer time series will be necessary to corroborate these findings and provide further insights into the novel patterns observed here. These early explorations suggest that size-based acoustic methods would bear fruitful insight into aquatic ecosystem management and conservation.
